# Supplementary figures and images for: Preparation and Characterization of Ibuprofen Containing Nano-Embedded-Microparticles for Pulmonary Delivery
Source: Pharmaceutics. 2023 Feb 6;15(2):545. doi: 10.3390/pharmaceutics15020545 (PMC9966045; doi:10.3390/pharmaceutics15020545)

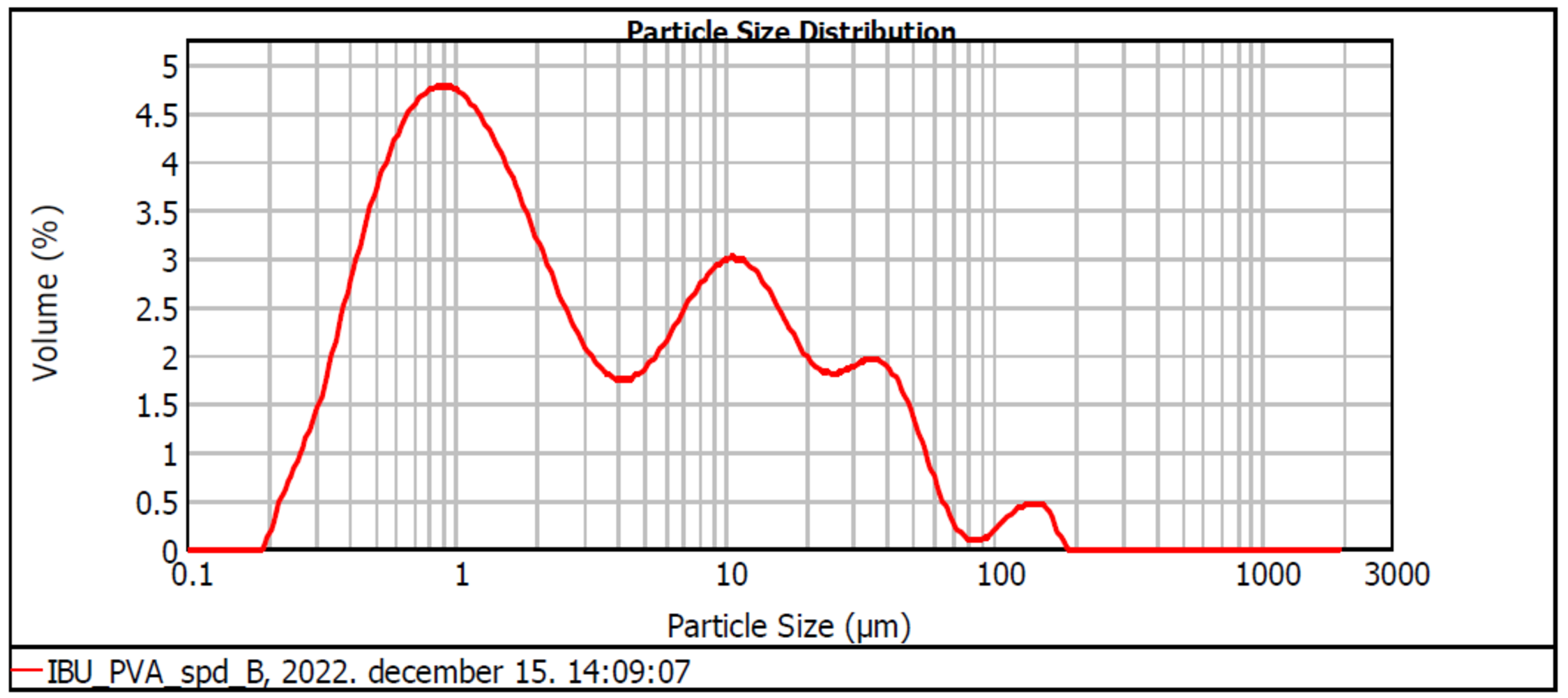

Supplement: Supplementary file 1 [file pharmaceutics-15-00545-s001.zip › Figure_S1.tif]

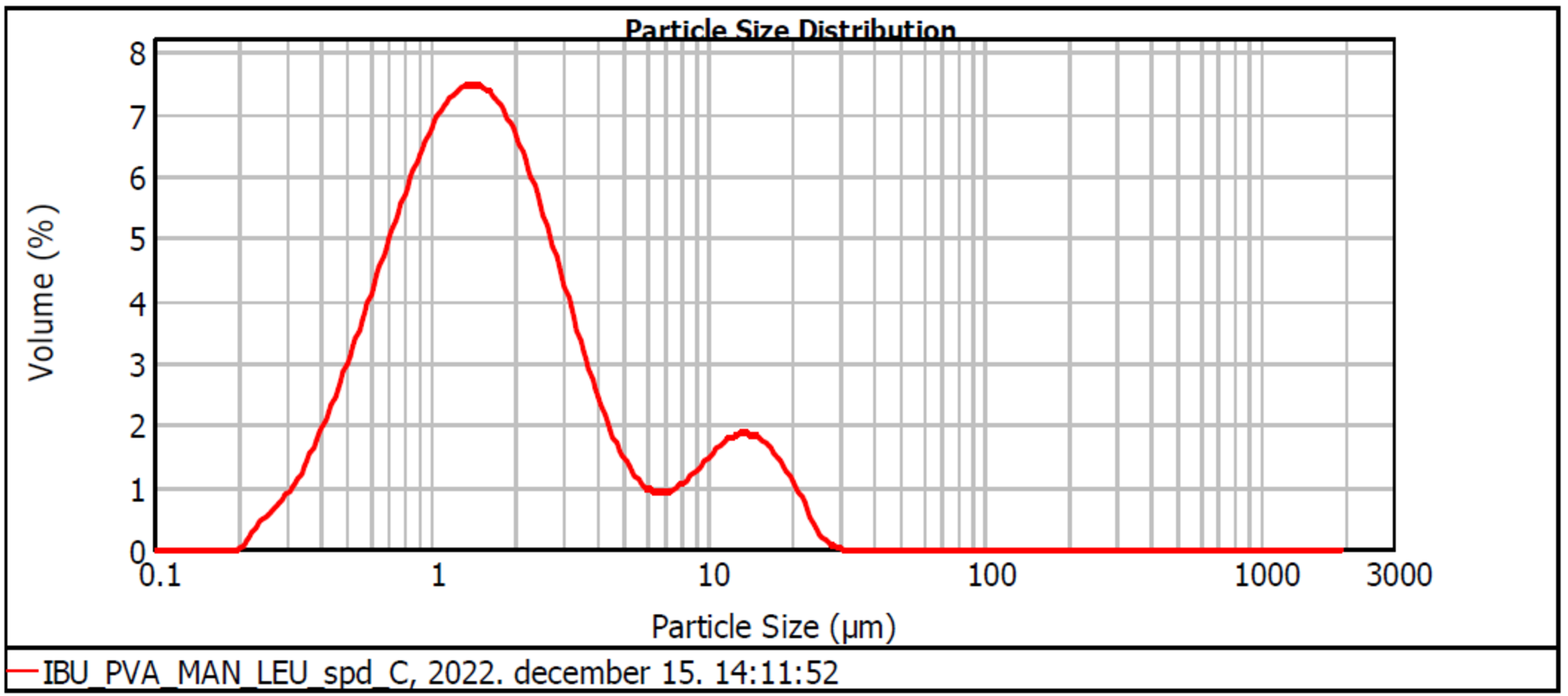

Supplement: Supplementary file 1 [file pharmaceutics-15-00545-s001.zip › Figure_S2.tif]
